# Can Tiger (TIG) Catheters Be a Solution to the Radial Artery Spasm (RAS) in Coronary Angioplasty? A Case-Based Report of Successful Reperfusion With the Use of 5-French (Fr) TIG Catheter and Literature Review

**DOI:** 10.7759/cureus.26334

**Published:** 2022-06-25

**Authors:** Zahid Khan, George Besis, Chetan Upadhyaya, Soon Neoh

**Affiliations:** 1 Acute Medicine, Mid and South Essex NHS Foundation Trust, Southend on Sea, GBR; 2 Cardiology and General Medicine, Barking, Havering and Redbridge University Hospitals NHS Trust, London, GBR; 3 Cardiology, Royal Free Hospital, London, GBR; 4 Cardiology, North Wales Cardiac Centre, Bodelwyddan, GBR

**Keywords:** st-elevation myocardial infarction, primary percutaneous coronary intervention, radial artery spasm, arterial spasm, coronary artery spasm, tiger catheter, drug eluting stents

## Abstract

Conventionally, during a primary percutaneous coronary intervention (PPCI), a diagnostic catheter is used to visualize the contralateral coronary system from the site of the acute occlusion. For that purpose, Judkins Right 4 (JR4) or Judkins Left 3.5 (JL3.5) diagnostic catheters are usually preferred, depending on the ECG findings. On the other hand, the use of a dedicated diagnostic catheter in the setting of PPCI is supported only by evidence extrapolated from coronary angiography on patients with stable coronary artery disease. We present a case of a 46-year-old patient who presented with ST-segment elevation myocardial infarction (STEMI) and underwent successful PPCI. A 6-French (Fr) radial sheath was placed in the right radial artery. Due to the presence of ST-segment elevation in both the inferior as well as in the anterior precordial leads, raising the possibility of a wrap-around left anterior descending (LAD) artery as the infarct-related artery, a 5-Fr Tiger (TIG) diagnostic catheter was initially used for cannulation of the left coronary system. The culprit lesion was identified in the proximal part of a small second right ventricular (RV) branch where it was 100% occluded with thrombus and the patient had a successful PPCI.

## Introduction

Coronary angiography is the best modality to measure coronary artery disease and is the recommended procedure in patients with acute heart attacks. Cardiac catheterization is an invasive procedure with serious risks associated with the procedure. Therefore, appropriate catheter size and equipment selection is crucial. Historically, 9- to 10-Fr guide catheters were used in the early years of percutaneous coronary intervention (PCI). However, lately, a 6-Fr catheter has become a standard for most PCI cases [1]. The pursuit of miniaturization illustrates the evolution of the guiding catheter and progress made to date to make angioplasty a safer procedure [1]. The development of smaller size catheters has not only resulted in a reduction in access site-related complications in the groin but has also enabled cardiologists to use radial artery access for PCI [2]. A major aim of any interventional cardiologist is to carefully plan the procedure and safely cannulate the vessel without any complications.

The first PCI through radial artery access was performed by Kiemeneij and his colleagues in 1992 and was termed trans-radial coronary intervention; it was found to be associated with significantly reduced procedure-related complications risk when compared to trans-femoral coronary intervention [3]. A major drawback of the trans-radial coronary intervention was reported to be radial artery spasm (RAS) and occlusion. Research has shown that although most radial artery occlusions are clinically silent, keeping the radial artery open is important for future coronary interventions and the concept of using smaller-sized catheters is based on the hypothesis that smaller size catheters are less likely to cause radial artery occlusion [1,4]. This is one reason why smaller size catheters such as 4- and 5-Fr are preferred over larger size catheters and it also helps to minimize the risk of causing RAS which makes access through radial artery extremely challenging.

Conventionally, during a primary percutaneous coronary intervention (PPCI), a diagnostic catheter is used to visualize the contra-lateral coronary system from the site of the acute occlusion. For that purpose, Judkins Right 4 (JR4) or Judkins Left 3.5 (JL3.5) diagnostic catheters are usually preferred, depending on the ECG findings. On the other hand, the use of a dedicated diagnostic catheter in the setting of PPCI is supported only by evidence extrapolated from coronary angiography on patients with stable coronary artery disease. We present a case of a 46-year-old patient who underwent emergency PCI through trans-radial access by using a Tiger (TIG) catheter after unsuccessful attempts to resolve pharmacologically severe coronary artery spasm.

## Case presentation

**Figure 1:**
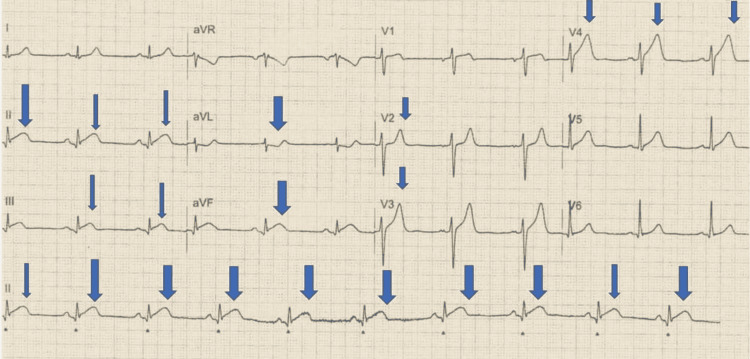
ECG shows inferior STEMI with reciprocal changes in anterolateral leads as shown by the arrows STEMI: ST-segment elevation myocardial infarction

A 46-year-old gentleman, with a past medical history of hypertension and a former smoker, presented with central chest pain at rest radiating to his left arm. His ECG confirmed the diagnosis of inferior ST-segment elevation myocardial infarction (STEMI) (Figure 1).

**Figure 2:**
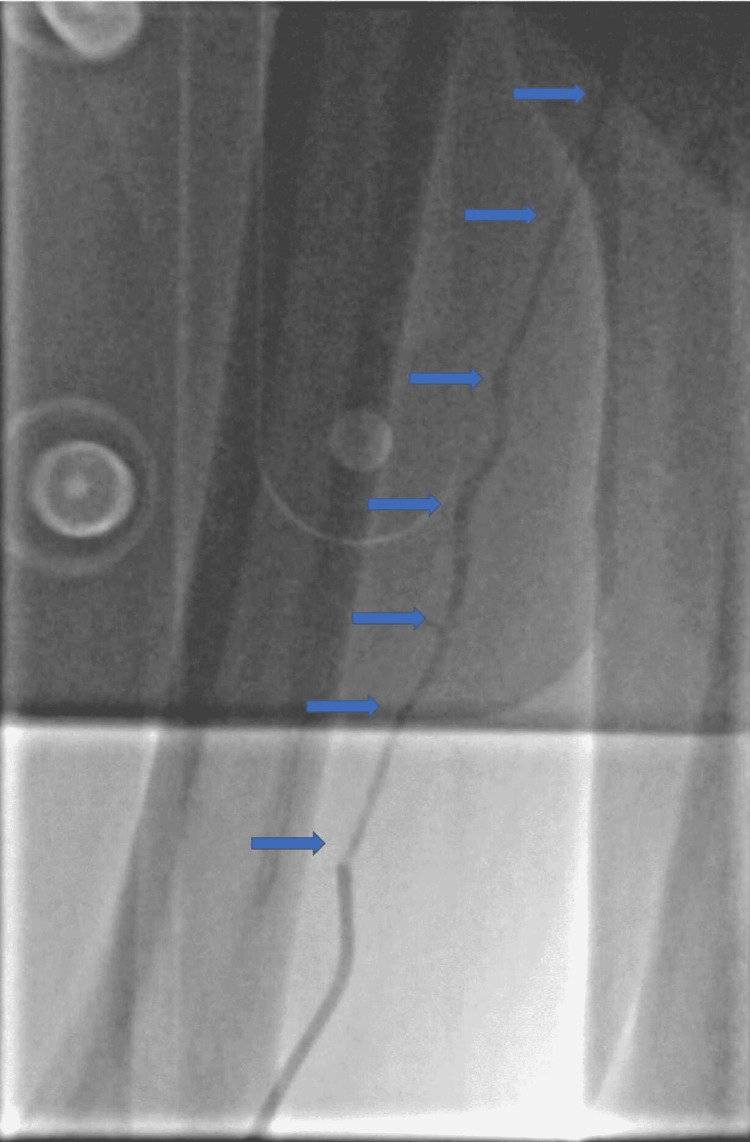
The right radial artery spasm (RAS) (arrows)

**Figure 3:**
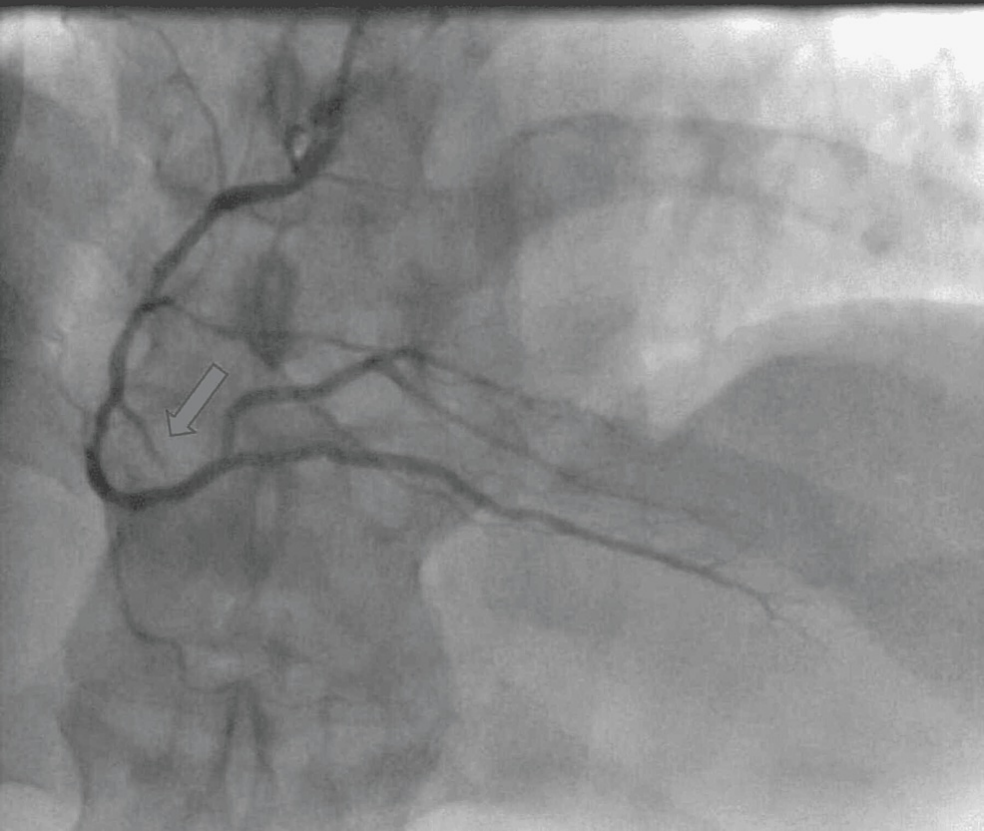
RCA cannulation with blocked small branch of the RCA (grey arrow) RCA: Right coronary artery

He was transferred to our center for primary PCI and was loaded pre-procedure with aspirin 300 mg and ticagrelor 180 mg as per local guidelines. A 6-Fr radial sheath was placed in the right radial artery. Due to the presence of ST elevation in both the inferior as well as in the anterior precordial leads, raising the possibility of a wrap-around left anterior descending (LAD) artery as the infarct-related artery, a 5-Fr TIG diagnostic catheter was initially used for cannulation of the left coronary system (Figure 2). The introduction of the 5-Fr TIG catheter over a standard 0.035-inch hydrophilic guidewire led to a significant radial spasm (Figure 3). He was given intra-arterial nitrate 200 mcg, IV midazolam 2 mg, and IV fentanyl 50 mcg.

**Figure 4:**
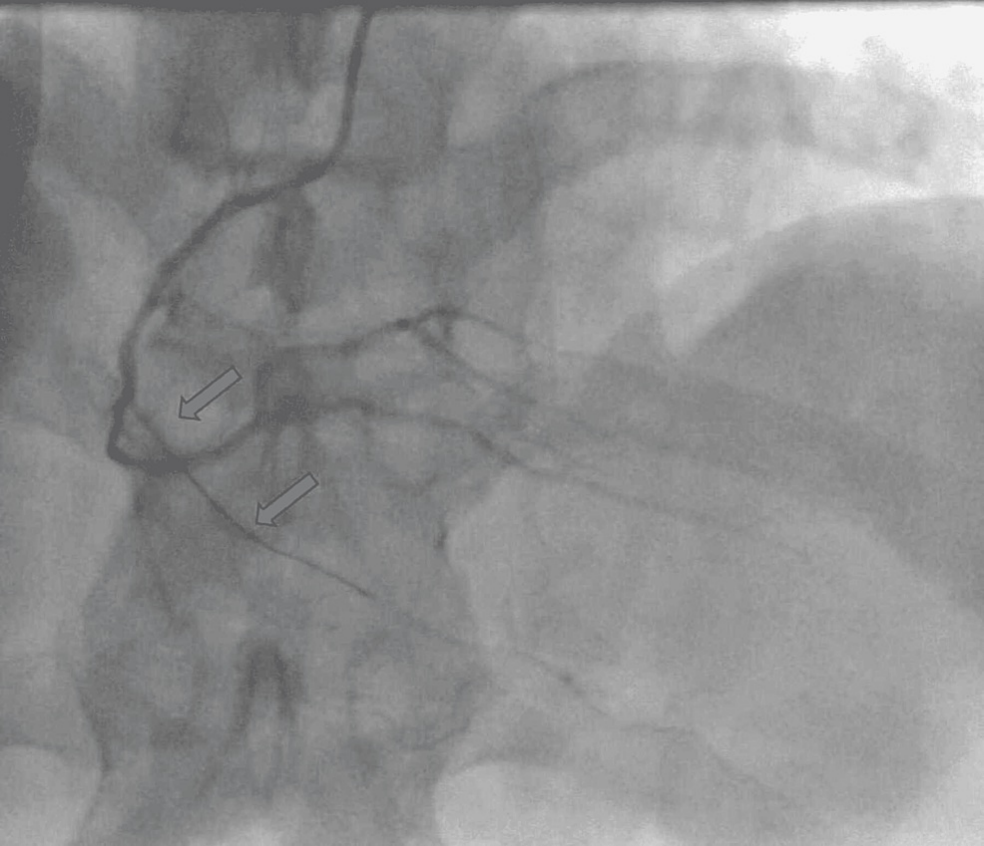
Occluded small branch of RCA after guidewire insertion RCA: Right coronary artery

The standard 0.035-inch wire was exchanged with a 0.014-inch Sion blue (Terumo, Shibuya City, Japan) wire, which successfully navigated past the spastic radial artery and enabled cannulation of the left coronary system. Due to the presence of a significant spasm which persisted after the left coronary system cannulation, we elected to cannulate the right coronary artery (RCA) to inform the selection of the appropriate type of guide catheter. The culprit lesion was identified in the proximal part of a small second right ventricular (RV) branch where it was 100% occluded with thrombus (Figure 4).

We commenced the patient on glycoprotein IIb/IIIa inhibitors (GP2b3a inhibitor) tirofiban with the view to treat the culprit artery medically if possible since it is a RV marginal branch. Also, the severe radial spasm would not allow us to advance a 6-Fr guide catheter past the radial artery and, thus, there was a high likelihood of conversion to femoral approach.

**Figure 5:**
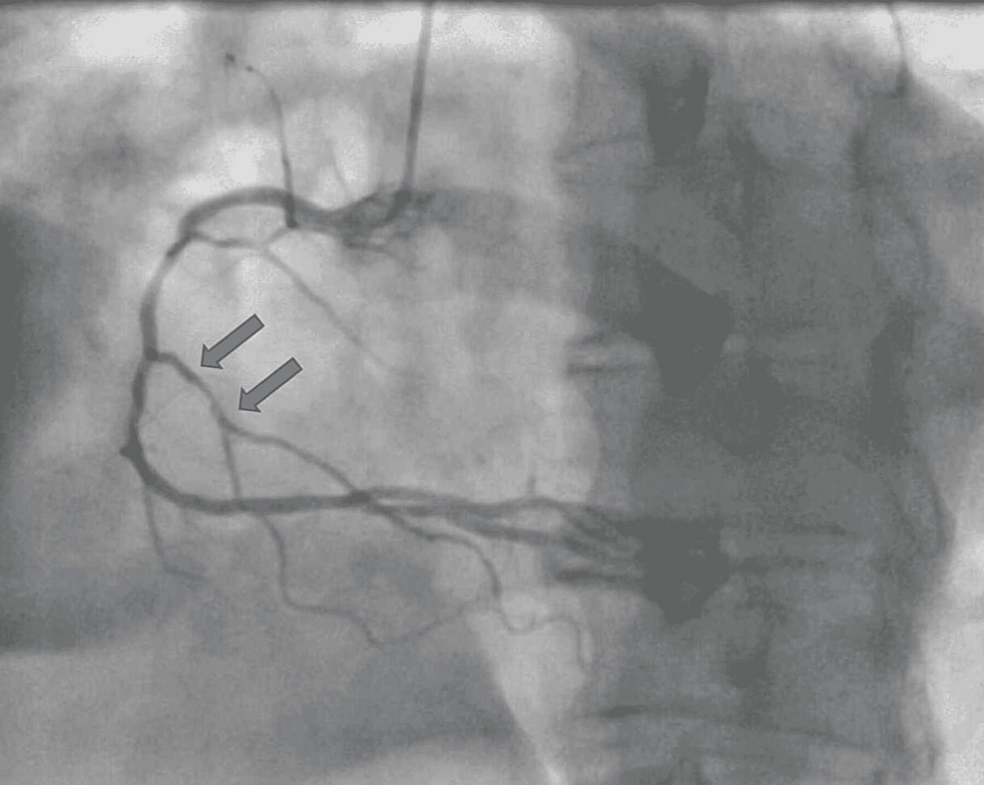
Coronary angiogram of the reperfused small branch of the RCA confirming TIMI 3 flow TIMI 3: Thrombolysis in myocardial infarction 3; RCA: Right coronary artery

Due to ongoing chest pain and ECG on screen still showing inferior ST elevation, we decided to perform coronary intervention with workhorse wire only via TIG catheter that is still engaged in the RCA. We passed the 0.014-inch Sion blue wire into RV marginal branch which re-established thrombolysis in myocardial infarction 3 (TIMI 3) flow successfully. Coronary angiography after TIMI 3 flow was established revealed a long RV branch of less than 2 mm diameter with proximal stenosis (Figure 5). Due to the small caliber of the vessel, the resolution of chest pain with the restoration of TIMI 3 flow and the normalization of the ECG we elected to avoid any balloon angioplasty or stenting.

## Discussion

The use of a single catheter for cannulation of both the right and left coronary system has been studied in the setting of elective diagnostic coronary angiography and was shown to have a similar success rate to JR4 and JL3.5 for cannulation for the right and left coronary system, respectively [4,5]. However, this approach has not been evaluated in a retrospective or prospective fashion in the setting of PPCI. In our case, successful cannulation of both left and right coronary ostium was achieved despite a severe RAS. Standard 12 lead ECG has modest diagnostic accuracy for identifying the infarct-related artery [6]. A dedicated radial catheter can correctly identify the culprit vessel, more importantly in the setting of inferior myocardial infarction. This approach may also have the potential advantage of enabling the selection of the most appropriate guide catheter, especially for RCA interventions (e.g., Amplatzer left (AL) catheter rather than JR4 in case of anterior origin of RCA ostium or when increased support is anticipated). Many techniques have been developed for the management of RAS, such as balloon-assisted tracking or the mother and child technique (use of a long 5-Fr diagnostic JR4 through a standard 6-Fr JR4 or JL3.5 guide catheter). In our case, the knowledge of the RCA anatomy and the identification of the infarcted-related artery as the second RV branch led us to attempt to establish TIMI flow using the TIG 5-Fr diagnostic catheter to facilitate faster reperfusion as we anticipated the small caliber of the vessel judging from the diameter of the patent first RV branch, which was not more than 2 mm in diameter. The use of a TIG catheter to re-establish flow in case of an acute anterior myocardial infarction has been previously reported [7]. To the best of our knowledge, this is the first case described in the literature where TIMI 3 flow in the right coronary system has been restored with the use of a diagnostic 5-Fr TIG catheter and it can be used to balloon the lesion.

Vascular complications are the most common set of post-catheterization problems. Therefore, the selection of the best access route and right catheter size is important to minimize access-related complications which can result in additional hospitalization and patients may require additional surgical procedures to repair the damaged vessels [7]. The selection of the most appropriate arterial access in turn depends on the operator's experience, patient anatomy, and procedural complexity [8]. Radial access is being used with greater frequency in most countries as a result of randomized control trials (RCTs) that showed lower bleeding risk, better patient outcomes, and lower hospital costs associated with radial access compared to femoral access [9]. The radial artery approach requires significant experience, and the technique has several advantages for patients such as earlier ambulation and reduced hemostasis complications when performed by an experienced operator. In obese patients, radial access can be used as an alternative approach to femoral technique with lesser complications risk. The average radial artery lumen is approximately 2 mm in size. Therefore, the choice of sheath-size selection is limited for any operator, and it is recommended that a sheath of 6-Fr or below should generally be used for radial approach to avoid arterial spasm [10].

As mentioned earlier that RAS is the most common complication of radial artery access and the common treatment methods utilized for RAS include various intra-arterial, intravenous, and topical medications such as calcium channel blockers and nitrates [11]. Unfortunately, debate still persists on topical medications alleviating RAS during transradial percutaneous procedures. A significant number of these patients are non-responsive to vasodilators, sedation, and arm warming techniques to relieve the RAS [12]. A common recommendation to minimize RAS includes ultrasound-guided vascular access to evaluate artery size and minimize the number of attempts, administration of a radial artery spasmolytic, use of an optimally sized hydrophilic arterial sheath, catheters, and fewer catheter manipulations [12].

Vessel tortuosity, lesion severity and length, lesion calcification, lesions located distal to a previously implanted stent, chronic total occlusion (CTO), length and structure of the stent, or poor guiding catheter support in dilated aortic roots are various characteristics associated with stent delivery failure [13]. The risk of failure is higher in transradial intervention and the main reason for failure is the lack of guide catheter support, and it is also one of the main reasons for conversion to the femoral approach in about 7% of cases. A large guide catheter may not be useful for deeper intubation despite offering good passive support [13]. In comparison, a smaller catheter may provide deeper intubation but poor backup support. Mother-Child Technique is one way to overcome this problem and it has the advantage of combining both and involves a longer "Child Catheter" introduced within a bigger and conventional length "Mother Catheter" and it provides superior trackability and stent deliverability even in tortuous vessels [13]. Two major studies reported the incidence of major complications related to coronary angiogram to be < 1% [14,15].

Langer et al. reported that the TIG catheter was associated with lesser complications compared to the Judkins catheter and the amount of contrast used and fluoroscopy time was less for TIG catheters. TIG catheters were associated with poor ostial instability in the right coronary artery compared to Judkins catheters. However, Judkins catheters were found to have a lesser frequency of poor ostial engagement in the left coronary ostium [16,17]. "The JUDGE study" was a two-center randomized controlled trial to compare TIG-II with Judkins 3.5L/4R catheters in coronary angiography via the transradial approach [18]. TIG-II catheter was associated with better imaging of the RCA, but poor imaging of the left coronary artery compared to the Judkins catheter. Additionally, the procedure time, fluoroscopy time and severe RAS rate were also significantly lower for TIG-II vs. Judkins group.

## Conclusions

In conclusion, coronary angiography is an invasive procedure with the risk of significant complications if an inappropriate size catheter is used; the risk is even higher in acute settings. It is important to select the right size catheter according to the patient; also, the risk of complications is lower in the hands of the expert operators. Smaller size catheters are generally associated with lower complications risk compared to bigger size catheters and in our case report, we managed to use a diagnostic TIG catheter to successfully achieve TIMI 3 flow. We believe that the use of TIG diagnostic catheter deserves further studies to establish if it is of benefit in minimizing reperfusion time in the setting of primary percutaneous intervention.
